# Climatic niche and flowering and fruiting phenology of an epiphytic plant

**DOI:** 10.1093/aobpla/plv108

**Published:** 2015-09-10

**Authors:** Narayani Barve, Craig E. Martin, A. Townsend Peterson

**Affiliations:** 1 Biodiversity Institute, University of Kansas, 1345 Jayhawk Blvd, Lawrence, KS 66045, USA; 2 Department of Ecology and Evolutionary Biology, University of Kansas, Lawrence, KS 66045, USA

**Keywords:** ERA data, Spanish moss, species distribution, *Tillandsia usneoides* L.

## Abstract

Species have geographic distributions constrained by combinations of abiotic factors, biotic factors and dispersal-related factors. Abiotic requirements vary across the life stages for a species; for plant species, a particularly important life stage is when the plant flowers and develops seeds. A previous year-long experiment showed that ambient temperature of 5–35 °C, relative humidity of >50 % and ≤15 consecutive rainless days are crucial abiotic conditions for Spanish moss (*Tillandsia usneoides* L.). Here, we explore whether these optimal physiological intervals relate to the timing of the flowering and fruiting periods of Spanish moss across its range. As Spanish moss has a broad geographic range, we examined herbarium specimens to detect and characterize flowering/fruiting periods for the species across the Americas; we used high-temporal-resolution climatic data to assess the availability of optimal conditions for Spanish moss populations during each population's flowering period. We explored how long populations experience suboptimal conditions and found that most populations experience suboptimal conditions in at least one environmental dimension. Flowering and fruiting periods of Spanish moss populations are either being optimized for one or a few parameters or may be adjusted such that all parameters are suboptimal. Spanish moss populations appear to be constrained most closely by minimum temperature during this period.

## Abstract (precis)

Each species passes through various life stages, and each life stage may have different requirements in terms of climate, soil, topography or other abiotic factors. The phenological stage is one such critical life stage in the plant life cycle. We examined the availability of optimal ecophysiological parameters in 22 years of high temporal climate data during the flowering and fruiting stage of an epiphytic plant, Spanish Moss, with a hemisphere-wide distribution. We used herbarium specimens to establish the flowering period. We found that most populations experience sub-optimal conditions in at least one environmental dimension and most populations are constrained by minimum temperature during the flowering/fruiting stage.

## Introduction

Restricted geographic distributions of species are often a consequence of some set of constraints in terms of abiotic requirements, needs in terms of biotic interactions and limitations to dispersal ability (Soberón 2007). All species have a life cycle (be it simple or complex), and each stage in that cycle may have different requirements in terms of climate, soils, topography, other abiotic factors and biotic requirements like food, competitors or mutualisms. Grubb (1977) defined four components of ecological niches of plants: the habitat niche, life-form niche, phenological niche and regeneration niche; much research has examined how regeneration niches may differ in different community assembly processes and how these various niches act in different life stages (Fowler 1988; Tilman 1990; Lavorel and Chesson 1995; Miller-Rushing and Primack 2008). Although several studies have used the regeneration niche concept to explore competition and understand rarity of species at local scales (Engelhardt and Anderson 2011; Ranieri *et al.* 2012), few studies have used the regeneration niche idea to understand species’ distributions in terms of their abiotic requirements at geographic scales (Pederson *et al.* 2004; Sweeney *et al.* 2006; Wellenreuther *et al*. 2012).

Phenological stages in plant life cycles comprise critical life stages, in which plants flower, produce seeds, grow or remain dormant (Bond and Midgley 2001; Silvertown 2004). Plants have presumably evolved to flower in seasons and at intervals that ensure maximal reproductive success (Amasino 2010). Considerable research has shown that plants sense and respond in complex ways to environmental cues such as shoots bending towards light and away from gravity (Garner 1933; Lang 1952; Bernier *et al.* 1993; Dennis *et al.* 1996). However, these factors have been investigated chiefly at local scales; at biogeographic scales, the question of whether phenology is optimized or not with respect to physiological responses to abiotic factors like temperature and precipitation remains little investigated (Engelhardt and Anderson 2011; Ranieri *et al.* 2012).

Here, we examine the timing of flowering and fruiting by Spanish moss (*Tillandsia usneoides* L.) populations across the species' broad geographic range in relation to availability of optimal physiological conditions (Barve *et al.* 2014). Physiological measurements have been made previously in year-long field experiments (Martin and Siedow 1981; Martin *et al.* 1981) to estimate intervals of climate-related parameters that are ideal for growth. We used herbarium specimen records of flowering and fruiting Spanish moss to identify population-specific flowering and fruiting periods and tested detailed environmental data for associations with minimum temperature, maximum temperature, relative humidity and rainless days requirements on a univariate basis, building on our earlier analyses of physiological limits in relation to climate across the range of this species (Barve *et al.* 2014). We use these analyses to test whether (i) all four parameters are at optimal physiological values as measured in previous studies during flowering periods and (ii) which physiological parameter(s) is (are) optimized during the flowering periods, if not all are optimized.

## Methods

### Study organism

Spanish moss (*T. usneoides*) is an epiphytic flowering plant of the family Bromeliaceae, distributed approximately between 38°N and 38°S latitude. It typically grows in warm and humid climates on trees or other supporting structures, such as power cables (Billings 1904; Garth 1964; Callaway *et al.* 2002). Spanish moss occurs over a broad elevational range (0–3300 m), and associations with atmospheric moisture content and temperature vary significantly according to elevation (Gentry and Dodson 1987; Kreft *et al.* 2004). The species does not occur at high elevations, which are apparently too cold for its persistence; indeed, its general natural history suggests that its distribution will prove to be highly constrained by climatic factors (Garth 1964), more or less in line with the ‘Hutchinson's dream’ scenario of Saupe *et al.* (2012).

Temperature, humidity and drought are known to affect growth and persistence of Spanish moss (Garth 1964; Martin and Siedow 1981; Martin *et al.* 1981; Martin and Schmitt 1989). A year-long field experiment (May 1978–May 1979) was performed by Martin *et al*. (1981) near Elizabethtown, NC, USA (78.594°W, 34.682°N); it found that Spanish moss growth is concentrated in summer months, with winter growth almost negligible. Martin *et al.* (1981) showed that CO_2_ uptake was maximal when daytime temperature is 5–35 °C; CO_2_ uptake was eliminated at or below 0 °C and at or above 40 °C. Kluge *et al*. (1973) also experimented on Spanish moss, with similar results regarding CO_2_ uptake; however, they used greenhouse-grown Spanish moss, and their experiment was carried out in the laboratory under constant temperature and humidity. Martin *et al*. (1985, 1986) assessed North Carolina Spanish moss populations with respect to irradiance effects on morphology and physiology, finding that Spanish moss responds to irradiance by adjusting physiology more than morphology. Garth (1964) showed that Spanish moss cannot survive in Georgia without periodic rainfall, even when water is supplied externally; he found that Spanish moss achieves optimal performance in terms of growth only with ≤15 consecutive rainless days. Martin *et al*. (1981) corroborated this latter result, with the additional information that CO_2_ uptake is minimal when Spanish moss is wet by rain, suggesting that Spanish moss requires some dry periods for persistence. Overall, then, these experiments identified four parameters that can be analysed at continental extents: minimum temperature ≥5 °C (Martin *et al.* 1981), maximum temperature ≤35 °C (Martin and Siedow 1981), night-time humidity ≥50 % (Martin *et al.* 1981) and ≤15 rainless days (Garth 1964).

### Input data

We collected information on flowering and fruiting periods of Spanish moss populations by examining herbarium specimens. We photographed 430 specimens in the collections of the Missouri Botanical Garden and 504 specimens from the New York Botanical Garden collections using a 16 megapixel Nikon P510 camera. We took four to five photographs per specimen to capture various details: one of the label to permit capture of associated data, one of the whole specimen and two to three zoomed photographs of flowers or fruits. In addition, we reviewed published floras for flowering dates, although most floras either do not offer sufficient detail about flowering period or do not provide precise locality information. Finally, we downloaded images from various herbaria listed on the Index Herbariorum site (http://sciweb.nybg.org/science2/IndexHerbariorum.asp) and others (http://herbarium.bio.fsu.edu and http://apps.kew.org/herbcat/navigator.do, 25 September 2015). Flowering and fruiting periods were assumed to be unimodal, so we filled temporal gaps for analyses of optimal physiological conditions. The temporal resolution of flowering and fruiting times was kept at months, so that imprecise date information (e.g. ‘April 1914’) could be incorporated, and quantity of relevant data maximized.

Information from specimen labels was digitized and stored in a Microsoft Access database. Some labels had geolocations in terms of latitude–longitude coordinates, whereas others had only textual locality information at various administrative levels. In the latter case, geolocations were attached to each record via queries in Google Earth. Overall, we were able to obtain information for 361 sites where both flowering date and geolocation information were available, which we used to profile flowering/fruiting periods at sites across the range of the species.

We examined how physiological thresholds are met (or not) for Spanish moss across the Americas within empirically documented flowering intervals over a 22-year period (January 1989–December 2010) following Barve *et al.* (2014). We used the ERA interim reanalysis climate data developed and supplied by the European Center for Medium-Range Weather Forecasts, which are based on a combination of models and observations, with three-hourly temporal resolution: every second datum is a forecast, whereas the other is a model result. We used only the model result data, thus coarsening the data from three- to six-hourly resolution, but retaining an impressively fine temporal resolution. The data set has a somewhat coarse native spatial resolution of 1.5° × 1.5° or ∼165 × 165 km grid square resolution at the Equator.

ERA data were downloaded from http://apps.ecmwf.int/datasets/data/interim_full_daily/ for the following parameters: minimum temperature at 2 m, maximum temperature at 2 m, mean temperature at 2 m, dew point temperature at 2 m and precipitation. The data are stored in NetCDF format (http://www.unidata.ucar.edu/packages/netcdf/index.html; Rew and Davis 1990); these data were manipulated and processed via the ‘ncdf’ package in R (Pierce 2011; R Core Development Team 2012). ERA interim data were processed to identify optimal and suboptimal areas and temporal duration of suboptimal conditions with respect to each physiological variable through time.

Overall, one hundred and thirty-six 1.5° grid squares held at least one Spanish moss record with flowering and fruiting information. As numbers of flowering records were not numerous with respect to so many grid squares, to improve data density, we coarsened the 1.5° grid to 3° grids only to characterize flowering periods, but climate data were kept at the original 1.5° resolution. We generated flowering and fruiting month ranges for each 3° grid square; we assumed single flowering/fruiting months in grid squares in which only single specimens were available, which may be a restrictive assumption in our analyses. We also generated non-flowering month data sets for each grid square for comparison; for example, for a grid square with a flowering/fruiting range of March–May, we generated the remaining 11 possible 3-month sequences for comparison. We identified the average flowering/fruiting month, flowering/fruiting season start and flowering/fruiting season end for each grid square. Average flowering/fruiting month was calculated as a weighted average based on number of flowering or fruiting specimens in each month.

### Data analysis

An R script was developed using the raster, ncdf and sp packages (Bivand *et al.* 2008; Pierce 2011; Hijmans and van Etten 2012) to calculate the percentage of time over the 22-year span of the data set that Spanish moss populations experienced optimal conditions with respect to the physiological thresholds described above. For minimum and maximum temperatures, the script checks the value of each variable across four daily observations; a grid square was marked as unsuitable for a day whenever two consecutive observations were outside the limit. For precipitation, whenever all four daily observations were 0 (i.e. no precipitation), it was considered as a day with no precipitation, and all consecutive sets of 15 days were checked; when any 15-day period had no precipitation, the grid square was considered as not suitable. For relative humidity, dew point temperature (*T*_d_) and mean air temperature at 2 m (*T*_a_) were used, and relative humidity was calculated as Rh = *e*_s_(*T*_d_)/*e*_s_(*T*_a_), or the ratio of saturation vapour pressure, *e*_s_, at dew point to that at air temperature, where *e*_s_ for any temperature *T* is given by *e*_s_(*T*) = 6.112 × e^(17.502 ×^*^T^*^/(240.97 +^*^T^*^))^ (Stull 1988). We identified grid cells as unsuitable whenever two consecutive observations fell below the humidity threshold. Likewise, we calculated the percentage of time that the grid square spent outside its optimal physiological thresholds within the flowering period for that grid square across the 22-year time span; for comparison, we also generated these percentages for all possible non-flowering periods of similar duration.

We ranked each grid square based on the percentage of time spent outside optimal values in flowering and fruiting periods and each other possible non-flowering period of similar duration. We calculated the rank of each of the observed flowering periods with respect to all other possible periods of the same duration as the number of time periods of non-flowering months that are more suitable. We used Kolmogorov–Smirnov test to compare distributions of the four variable ranks.

Based on ranks of each grid square for each of the variables, we compared the actual flowering period with the optimum flowering and fruiting periods with respect to those variables. This distance was calculated as a Euclidian distance from an optimal rank of 1 for each of the variables. This Euclidian distance is normalized to a scale of 0–1, such that small distances indicate optimal flowering and fruiting periods for a population, whereas large distances suggest that the population flowers during suboptimal periods. We averaged this distance across all four physiological parameters and mapped these deviations from optimum. We tested for effects of number of herbarium specimen records in each grid square to these optimum distances.

## Results

We were able to assemble 361 flowering or fruiting records for Spanish moss across the species' range. Although records concentrated in the US portion of the species' range (159 records, or 44 %), the remaining 202 (56 %) records came from Latin America. Although densities of Latin American points were low at finer spatial resolutions (i.e. most grid squares had single or no flowering-period records), 3° spatial resolution was sufficient to create 83 grid squares, within which we had 1–28 flowering/fruiting records.

**Figure 1.**
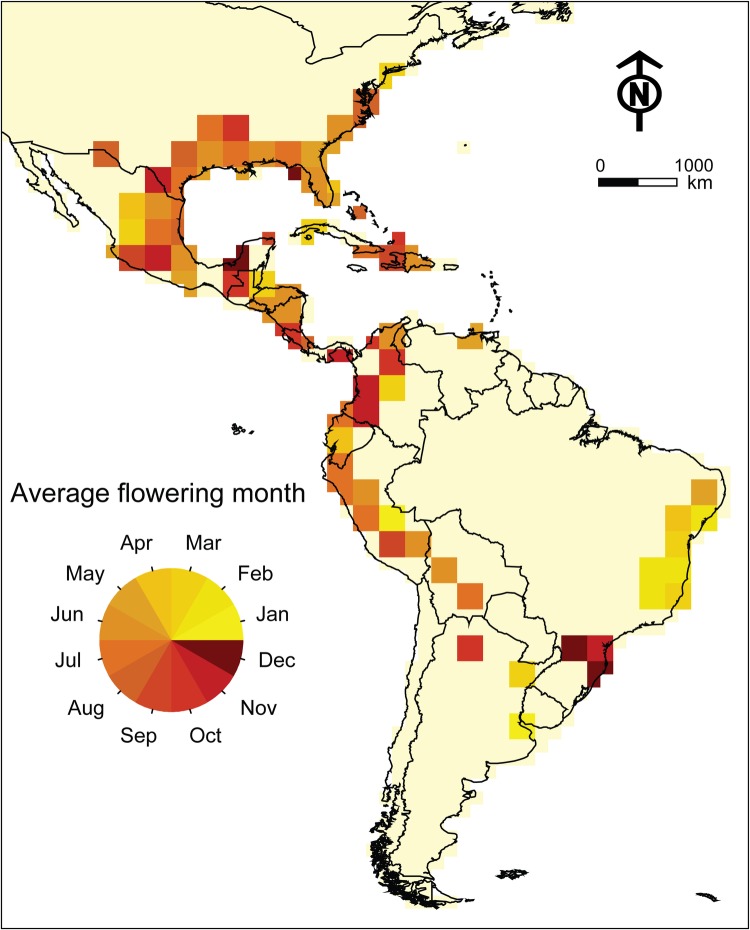
Average flowering month of Spanish moss populations across the Americas calculated as weighted average of flowering or fruiting specimens recorded from each grid square.

Average flowering and fruiting month of Spanish moss populations across the species' range is shown in Fig. 1. The flowering and fruiting periods in eastern Brazil were November–April, while the flowering and fruiting periods in western South America were June–September, with a few exceptions extending to October–November. The flowering period in the USA and Mexico was May–September, with a few exceptions in November–December. Because our identification of flowering and fruiting month(s) was in some sense dependent on numbers of specimens available, we suspect that insufficient data density may be driving the exceptions.

**Figure 2.**
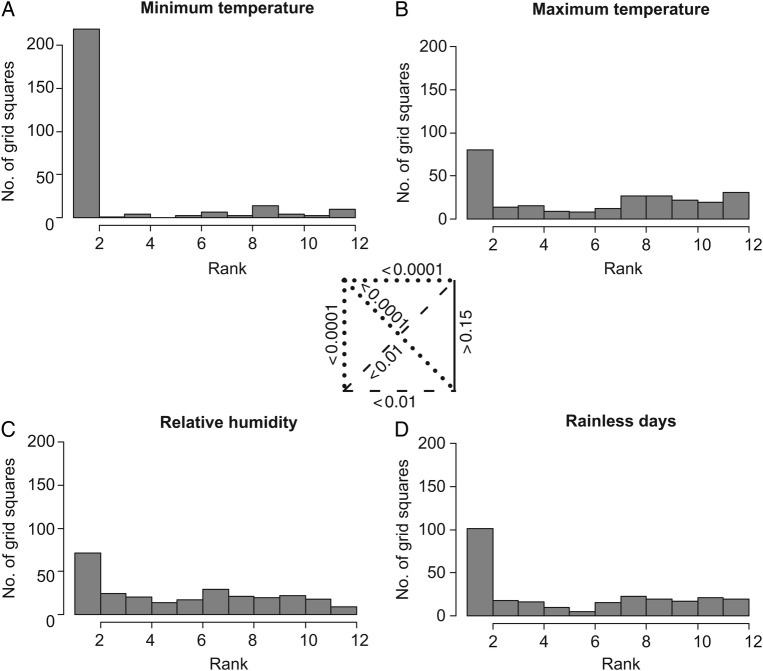
Histogram of ranks based on how long populations in each grid square are outside optimal conditions for each of the four parameters during their flowering and fruiting periods. The box at the centre shows results of Kolmogorov–Smirnov tests for comparison of distributions. Dotted lines indicate highly significant difference, dashed lines significant differences and continuous lines non-significant difference.

Flowering periods invariably fell in time periods in which least one physiological parameter was optimal in a grid square. The ‘optimal’ parameter was generally minimum temperature: that is, in 212 of 262 cases, flowering and fruiting periods coincided with months in which minimum temperature was within optimal ranges in at least 70 % of grid square–month combinations. Very few populations experienced minimum temperatures below the 5 °C criterion during their respective flowering and fruiting periods (Fig. 2A). The remainder of Fig. 2 suggests that flowering and fruiting periods depend less critically on parameters like maximum temperature, rainless periods or relative humidity. Ranking months by their optimality for each parameter, a Kolmogorov–Smirnov test revealed that distributions of ranks for minimum temperature were significantly lower than those for the other three factors (*P* < 0.0001, Table 1). The distribution of ranks among grid squares did not differ between maximum temperature and rainless days, whereas ranks of rainless days versus relative humidity showed the latter as significantly more optimal (*P* < 0.001, Table 1).

**Table 1.** Result of Kolmogorov–Smirnov test to compare distributions of ranks within four variables, viz, minimum temperature, maximum temperature, rainless days and relative humidity. Upper diagonal are the *P*-values and lower diagonal represents the test statistics.

*P*-valueTest statistics	Minimum temperature	Maximum temperature	Rainless days	Relative humidity
Minimum temperature		<0.0001	<0.0001	<0.0001
Maximum temperature	0.5417		>0.1	<0.005
Rainless days	0.4886	0.1023		0.006
Relative humidity	0.6364	0.1402	0.1477

**Figure 3.**
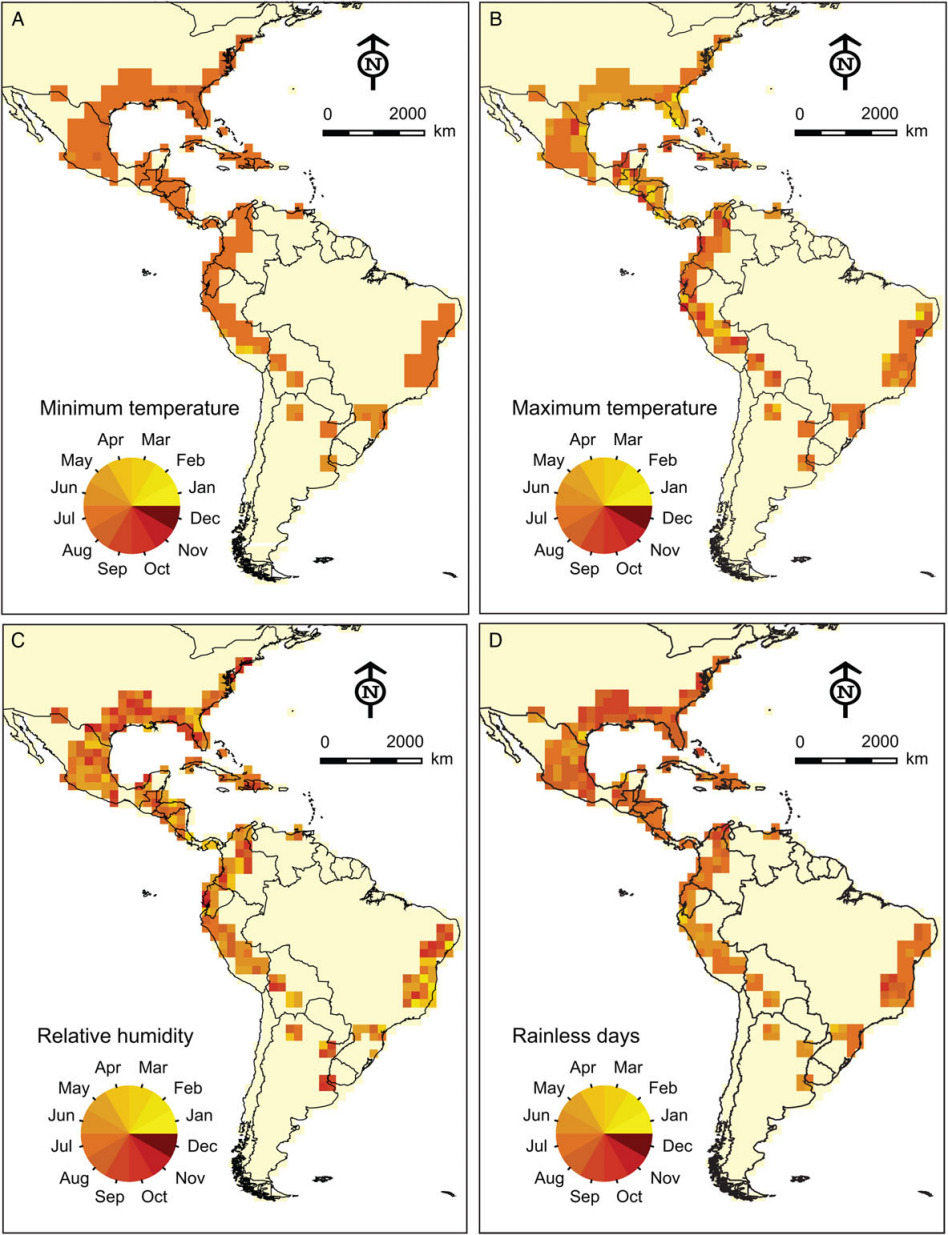
Optimal flowering and fruiting months for Spanish moss populations based on each physiological parameter in isolation.

We identified the optimal month for each pixel across the Americas in terms of each dimension of Spanish moss physiology. Figure 3A shows the optimal flowering and fruiting months for minimum temperature, which centred on July at the northern limit of the distribution, but in January–April at the southwestern distributional limit. However, for maximum temperature, the average expected flowering/fruiting month was February–April at the northern limit of distribution and April–August at the southwestern limit. Similar variation can be seen for relative humidity and rainless days: in short, no pixel had any period in which all four physiological parameters were in optimal states for flowering and fruiting to occur.

**Figure 4.**
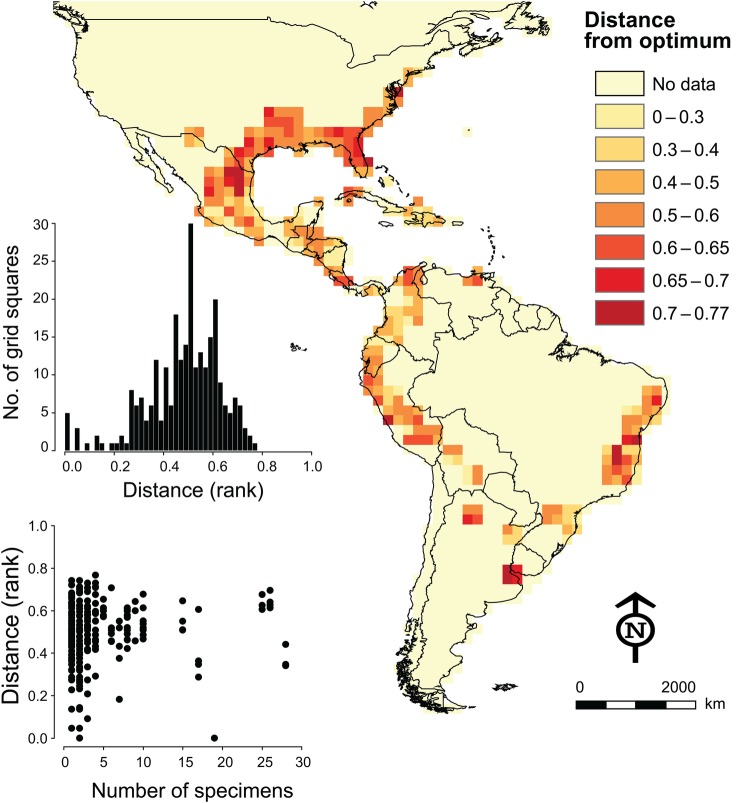
Map of Euclidean distances from observed conditions for Spanish moss populations to the best available across the species' distribution. Inset shows a frequency histogram of distances in grid squares (top) and relationship to numbers of specimens on which distance calculations were based (bottom).

To explore how far observed flowering and fruiting months departed from optimal months, we calculated average Euclidean distance in four-dimensional parameter space, ranking months by their suitability, standardizing each dimension to a range of 0–1 (thus creating an index of distance that has rather unclear units but that is useful for visualization) and counting ranks as greater distance from optimal conditions (Fig. 4). Most populations (46 %) showed flowering and fruiting periods with ranked Euclidean distances of ≤0.5. Only a few pixels were under extremely bad conditions and these higher-distance populations were arrayed at the extremes of the distribution (Fig. 4). We tested whether number of available flowering/fruiting specimens affected these latter results (see scatterplot inset in Fig. 4) but found no effect of sample size on distance to optimal month.

## Discussion

In overview, we found that Spanish moss populations appear to ‘tune’ their phenological niches such that they experience optimum minimum temperatures for most of their respective flowering and fruiting periods. Among populations analysed, flowering and fruiting periods of ∼76 % of Spanish moss populations experienced optimal minimum temperatures when compared with other time periods throughout the year. Conversely, Spanish moss populations appear to flower and fruit without much consideration of optimality of maximum temperature or relative humidity optimality, though rainless days do have some importance.

Numerous recent studies have documented shifts in flowering and fruiting season as a consequence of climate change (Molau *et al.* 2005; Miller-Rushing and Primack 2008; Telemeco *et al.* 2013). Veriankaitė *et al*. (2010) explored optimum temperatures for flowering and fruiting by comparing air temperatures in climate models with long-term flowering data. However, for this study, we took advantage of known optimum physiological parameters (Martin and Siedow 1981; Martin *et al.* 1981; Martin and Schmitt 1989), so we could explore the degree to which Spanish moss flowering and fruiting periods coincide with months presenting optimal physiological conditions for growth.

Phenological differences are well documented as functions of elevation and latitude (Ruml *et al.* 2011; CaraDonna *et al.* 2014). However, we generated our phenological information from herbarium specimens: few had elevation information, so effects of elevation on flowering phenology cannot be examined particularly in light of the coarse spatial resolution of our weather data. Clearly, as the climate data are coarsened and averaged over broader extents, such details average out in the climate and become invisible to our analyses, as was noted in our previous analyses (Barve *et al.* 2014). Our analyses may also be compromised by our rather coarse characterization of flowering and fruiting periods (i.e. to month) and by our filling of temporal gaps in flowering periods under the assumption of a single, continuous flowering and fruiting period for each population.

For Spanish moss, we observed that flowering phenology does not generally depend much on maximum temperature. Rather, minimum temperature appears to play a major role (Fig. 2). Comparisons with every other period of similar length in the year for each location suggested that Spanish moss flowering and fruiting periods are moulded such that flowering populations experience optimal minimum temperatures. Hence, an interesting challenge for long-term studies would be to test whether Spanish moss flowering and fruiting advances temporally in relation to rising minimum temperatures, rather than other climate characteristics of warming climates.

In our trade-off maps (Figs 2 and 4), we see that most Spanish moss populations show trade-off distances of 0.5 or less; nonetheless, some populations showed more substantial trade-off distances. Spanish moss populations under such suboptimal conditions likely face challenges to long-term persistence, suggesting that optimality of conditions in flowering period represents a constraint on Spanish moss geographic distributions. Although it is hard to say whether or to what degree climate change will change the geographic distributional potential of Spanish moss, Spanish moss may not flower and produce seeds successfully if climate change takes populations too far from optimal conditions. Even under present-day conditions, our approach can be used to locate where populations of the species will be under particular physiological stress.

## Conclusions

We analysed high-temporal-resolution (6-h resolution) climate data over a 22-year span to assess the availability of optimal conditions during flowering and fruiting periods of Spanish moss populations. Our results indicate that Spanish moss populations appear to flower and produce fruit seasonally such that populations experience optimum minimum temperatures. Our finding also shows that the least optimal conditions are experienced by populations along the fringes of the species' distribution. This research is novel in that we used herbarium specimens to assign flowering period to populations, that actual physiological measurements were used to assess optimality of conditions and that high-temporal-resolution weather data were used to provide a near-real-time view of the environmental conditions experienced by the species.

## Sources of Funding

This research was supported partly by Microsoft research grant to A.T.P., and N.B. was supported by grants from the Association of Women Geographers and Panorama Fund from the University of Kansas, Biodiversity Institute.

## Contributions by the Authors

N.B. and A.T.P. conceived the ideas, N.B. collected and analysed the data, N.B. led the writing and all authors contributed to the writing process.

## Conflict of Interest Statement

None declared.
